# Peptidomics and proteomics data of oxidised peptides from *in vitro* gastrointestinal digestion of chicken breast exposed to chlorpyrifos

**DOI:** 10.1016/j.dib.2020.106160

**Published:** 2020-08-08

**Authors:** Johana Márquez-Lázaro, Leticia Mora, Darío Méndez-Cuadro, Erika Rodríguez-Cavallo, Fidel Toldrá

**Affiliations:** a Analytical Chemistry and Biomedicine Group. Pharmaceuticals Sciences Faculty, Campus of Zaragocilla. University of Cartagena, 130015 Cartagena, Colombia.; b Instituto de Agroquímica y Tecnología de Alimentos (CSIC), Avenue Agustín Escardino 7, 46980, Paterna (Valencia), Spain.

**Keywords:** Chicken breast, chlorpyrifos, Oxidation, Digestion, Peptidomics, Proteomics

## Abstract

Chlorpyrifos (CPF) is an organophosphorus pesticide used in poultry to prevent and/or kill insects and such as preserving agents of poultry feed. Exposure continues to CPF can promote its accumulation at trace concentrations in animal tissue. The toxicological effects of these residues (carcinogenicity, genotoxicity, and neurological disorders) have been related to oxidative stress. Nevertheless, it is still unknown if these trace concentrations might promote oxidative stress in muscle proteins since chicken meat proteins are susceptible to undergo oxidation reactions. Moreover, protein oxidation has been related to a decrease in the nutritional value in of meat.

To investigate the oxidative effect of CPF on chicken breast proteins, peptidomics and proteomics analysis were used. For this, chicken breast samples were exposed to CPF and subjected to simulated gastrointestinal digestion. The identification of oxidized peptides from digested and undigested proteins were performed by LC MS/MS (nanoESI qQTOF). Prior to mass analyses undigested proteins were trypsinated. Data were analysed using MASCOT and ProteinPilot v 4.5 software. In this study, 90 and 107 oxidized peptides from digested proteins of control and exposed samples were identified, respectively. These peptides corresponding to 12 oxidized proteins. Meanwhile, 260 and 324 oxidized peptides from undigested proteins (control and exposed samples) were identified, which corresponding to 19 and 17 proteins, respectively. Collagen was protein more susceptible to oxidation promoted by chlorpyrifos in digested and undigested proteins. Identification of these oxidized proteins from simulated digestion provides an important insight about the impact of substances like certain veterinary drugs at trace concentrations on the nutritional value of chicken breast meat.

**Specifications Table**

**Table:** 

Subject	Food Science
Specific subject area	Meat science, peptidomics, proteomics, oxidized proteins
Type of data	Table Figure
How data were acquired	Mass spectrometer nanoESI qQTOF (5600 TripleTOF, ABSCIEX, Washington, D.C., United States).
Data format	Raw Analyzed Filtered
Parameters for data collection	Chicken breast samples were exposed to chlorpyrifos at 100 µg.Kg^−1.^ Then, control and exposed samples were carried out to simulated digestion. Peptides from digested and undigested proteins were identified by mass spectrometry.
Description of data collection	Oxidized peptides from digested and undigested proteins of both control and exposed chicken breast samples were identified.
Data source location	Instituto de Agroquímica y Tecnología de Alimentos Paterna/Valencia Spain
Data accessibility	With the article.
Related research article	J. P. Márquez-Lázaro, L. Mora, D. Méndez-Cuadro, E. Rodríguez-Cavallo, F. Toldrá, In vitro oxidation promoted by chlorpyrifos residues on myosin and chicken breast proteins, Food Chemistry, Volume 326, 2020, 126922, ISSN 0308-8146, https://doi.org/10.1016/j.foodchem.2020.126922. (http://www.sciencedirect.com/science/article/pii/S0308814620307846)

**Value of the Data**

• The data contribute to the understanding of the oxidative impact of chlorpyrifos residues on meat proteins.
• The raw data provides information about amino acids and proteins susceptible to suffer oxidation promoted by chlorpyrifos during digestion.
• The data allows to compare the oxidative changes in the proteome of digested proteins from control and exposed samples.
• These data are a useful resource to evaluate the impact of veterinary substances on edible products of animal origin.

## Data Description

### Peptidomics analyses

**Fig. 1:**
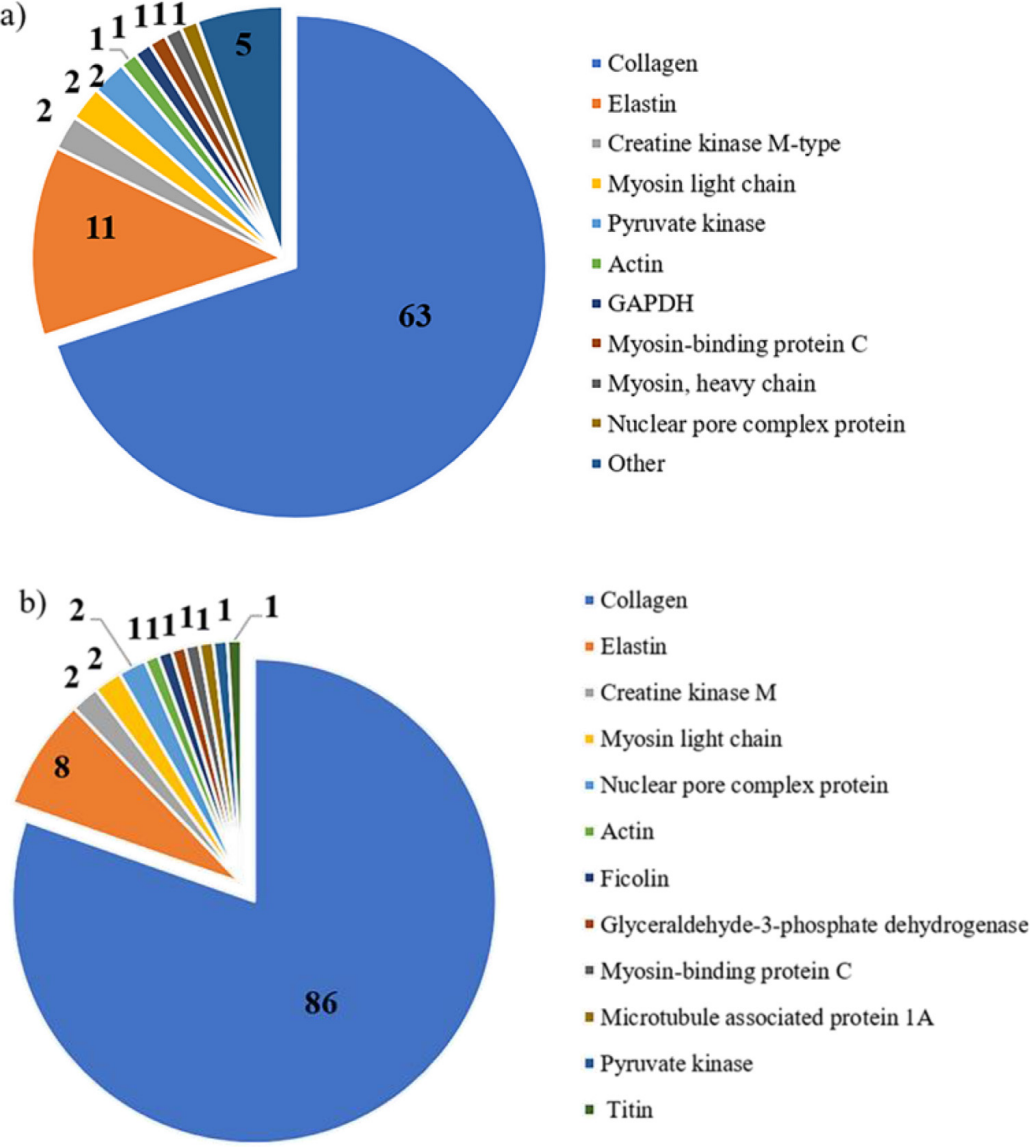
Distribution of the oxidized peptides identified by nLC-MS/MS after digestion according to their protein of origin: a) control and b) exposed samples.

This dataset contains raw and processed data of LC-MS/MS analysis from digested proteins of control and exposed samples. Supplementary Tables S1 and S2 give raw data of all peptides and proteins that were identified by mass spectrometry. The identified peptides from digestion with ≥80% confidence are shown in Supplementary Table S3 and S4 (Control and exposed samples, respectively). Moreover, the names of uncharacterized proteins are provided according the search done in protein BLAST [1]. The search was restricted to *Chordata* organism (taxid:7711). Supplementary Tables S5 and S6 show the oxidized peptides that were identified according each protein as well as oxidation site in peptide sequence. The oxidized peptides number were obtained by each proteins and sample type (control and exposed) are summarized in Fig 1. Collagen was proteins more susceptible to oxidation in both sample type. However, the number of oxidized peptides was highest in exposed samples than control (86 vs 63 respectively). In control, titin was the second protein with the highest number of oxidized peptides (Fig. 1a), while, elastin was that in the exposed samples (Fig. 1b).

### Proteomics analyses

Data contains information obtained of undigested proteins (control and exposed samples) carried out through trypsin digestion. Supplementary Tables S7 and S8 provides raw data of the undigested proteins were identified in control and exposed samples, respectively. The raw data of identified peptides and their posttranslational modifications are shown in Supplementary Tables S9 and S10 (Control and exposed samples, respectively). Identified oxidized peptides and their respective protein from control and exposed samples are summarized in Supplementary Tables S11 and S12, respectively. Also, in protein BLAST (Chordata organism, taxid:7711) the names of uncharacterized proteins were searched [1].

## Experimental Design, Materials and Methods

### Sample preparation and contamination

Chicken breast (*Pectoralis muscle*) was obtained from a local market in Valencia, Spain. Then, it was cleaned by eliminating adipose and connective tissue. Then, it was homogenized. Homogenized sample was divided in two portions of two and five grams in order to extract total proteins and to prepare the *in vitro* gastrointestinal digestion assay, respectively. Then, samples were exposed to CPF at 100 µg.Kg^−1^, followed by vortex during 30 s and 1 h incubation in the darkness and ambient temperature (24°C). Samples without CPF were used as control. Nitrogen gas (N_2_) was placed on each sample tube for minimize the oxidation promoted by oxygen in the incubation process.*In vitro* gastrointestinal digestion

The *in vitro* gastrointestinal digestion of samples was performed according to the procedure described by Minekus et al [2] with minor modifications [3]. The digestion was developed in a Carousel 6 Plus Reaction Station, (Radleys, UK). Regarding the gastric phase, samples were diluted in 10 mL of HCl 0.01 N, pH 3.0. Porcine pepsin and CaCl_2_ were added to reach a concentration of 2,000 U.mL-1 and 50 mM, respectively. Gastric digestion was done during 2 h at 37°C and constant stirring, the gastric digestion was stopped by adjusting pH to 7.0 (NaOH 1 M). Regarding the intestinal phase, a mixture of trypsin, chymotrypsin, pancreatic α-amylase, pancreatic lipase and bile extract to get a final concentration of 100 U.mL^−1^, 25 U.mL^−1^, 200 U.mL^−1,^ 2000 U.mL^−1^ and 10 mM, respectively. CaCl_2_ was also added to reach a final concentration of 50 mM, and sample was incubated for 2 h at 37°C. The intestinal digestion was stopped heating during 2 min at 95°C. Finally, proteins were precipitated adding 3 volumes of ethanol and maintaining the sample at 4°C during 20 h. Then, the precipitated was separated by centrifugation at 12,000 rpm and 4°C for 10 min and kept for further analysis. The supernatant was dried in a rotatory evaporator and lyophilized for MS/MS analysis. The assay was performed in duplicate.

Later, the precipitated proteins were dissolved in NH₄HCO₃ 50 mM with Tris buffer 10 mM, pH 8.55 and protein content was determined by BCA assay (Smith et al., 1985) in order to subject the samples to trypsin digestion. Thus, 100 µg of undigested protein were reduced with 2 µL of 45 mM DTT at 50°C for 15 min and alkylated with 2 µL of 50 mM IAA in the darkness and ambient temperature. The IAA excess was eliminated with 2 µL of 45 mM DTT. Finally, the proteins were digested using trypsin in a ratio protein/enzyme 1:50 (w/w) and incubated overnight at 37°C. The samples were analysed by MS/MS.

### Identification of peptides by liquid chromatography and mass spectrometry in tandem

Trypsinated proteins and peptides obtained after simulated gastrointestinal digestion were analyzed in a nanoESI qQTOF (5600 TripleTOF, ABSCIEX) instrument. Thus, samples were diluted in 50 µL of TFA 0.1% in ACN 2%. 5 µL of sample was loaded onto a trap column (NanoLC Column, 3 µm, C18-CL, 350 µm x 0.5 mm; Eksigent) and concentrated using TFA 0.1% as mobile phase at a flow of 3 µL.min^−1^ during 5 min. The peptides were then loaded onto an analytical column (C18-CL Nikkyo, 3 µm, 75 µm x 12 cm) equilibrated in ACN 5% FA 0.1% (formic acid). The elution was done using a linear gradient of 5 to 35% B in A for 60 min at a flow rate of 300 nL.min^−1^, where A is bidistilled water with 0.1% FA and B is ACN with 0.1% FA,. Analysis was carried out in DDA mode. Survey MS1 scans were acquired from 350–1250 *m/z* for 250 ms. The quadrupole resolution was set to ‘UNIT’ for MS2 experiments, which were acquired 100–1500 *m/z* for 50 ms in ‘high sensitivity’ mode. Main conditions in the mass spectrometry analysis were analysis of ions from 1+ to 5+, with a minimum intensity of 70 cps. Up to 25 ions were selected for fragmentation after each survey scan. Dynamic exclusion was set to 15 s. The system sensitivity was controlled with 2 fmol of 6 protein standards (LC Packings).

### Data analysis

The analysis of the obtained spectra was done using ProteinPilot v 4.5 search engine. ProteinPilot default parameters were used to generate a peak list directly from the mass spectrometry instrument. The Paragon algorithm [4] of ProteinPilot v 4.5 was used to search the Uniprot_Aves (Nov 2018) database using none enzyme as parameter. The posttranslational modifications such as oxidation of methionine and proline amino acids were determined automatically by Paragon algorithm.

## Declaration of Competing Interest

The authors declare that they have no known competing financial interests or personal relationships which have, or could be perceived to have, influenced the work reported in this article.

## Appendix Supplementary materials

Image, application 1

Image, application 2
